# Immunotherapy as a Promising Treatment for Prostate Cancer: A Systematic Review

**DOI:** 10.1155/2017/4861570

**Published:** 2017-10-03

**Authors:** Marlena Janiczek, Łukasz Szylberg, Anna Kasperska, Adam Kowalewski, Martyna Parol, Paulina Antosik, Barbara Radecka, Andrzej Marszałek

**Affiliations:** ^1^Department of Clinical Pathomorphology, Collegium Medicum in Bydgoszcz, Nicolaus Copernicus University in Torun, Torun, Poland; ^2^Department of Clinical Oncology, Oncology Center in Opole, Opole, Poland; ^3^Department of Oncologic Pathology and Prophylactics, Poznan University of Medical Sciences and Greater Poland Cancer Center, Poznan, Poland

## Abstract

Prostate cancer treatment is currently based on surgical removal, radiotherapy, and hormone therapy. In recent years, another therapeutic method has emerged—immunological treatment. Immunotherapy modulates and strengthens one's immune responses against cancer. Neoplastic cells naturally escape from the control of the immune system, and the main goal of immune therapy is to bring the control back. Satisfying outcomes after treatment of advanced melanoma and lung cancer suggest a great potential of immunotherapy as an approach for other tumors' treatment, especially in patients primarily introduced to palliative care. After initial clinical trials, immunotherapy seems to have different side effects than chemotherapy. Prostate cancer was the first neoplasm in which a specific vaccine significantly improved survival. There is a tremendous potential for synergistic combinations of immunotherapy with conventional cancer treatments. A combination of several drugs or methods can be a key in radical treatment of metastatic prostate cancer as demonstrated by preliminary studies.

## 1. Introduction

Prostate cancer (PC) is the second leading cause of cancer death in men, behind only lung cancer [1]. The American Cancer Society estimates that over 180,000 new cases of prostate cancer will be diagnosed in 2016 [2]. Additionally, a review of almost 800,000 cases revealed that the annual incidence of metastatic prostate cancer increased significantly in recent years [3]. Prostate cancer usually does not show any signs until it has progressed to an advanced stage. Prostate-specific antigen (PSA) has been used as a tumor marker for many years; however, the US Preventive Services Task Force (USPSTF) recommends against any routine PSA-based screening for prostate cancer [4]. Researchers pointed the possibility of overtreatment based on the aforementioned screening method with an increasing risk of side effects. Standard treatments of PC include surgical removal, radiation, and hormone therapy. In the last years, immunotherapy as an alternative method has gained increasing interest. Immunotherapy appears promising and probably will improve therapeutic strategy for patients with PC, resulting in increased quality and quantity of life. Immunotherapies fall into three categories such as checkpoint inhibitors, cytokines, and therapeutic cancer vaccines [5] (Table 1). There are numerous clinical trials on immune checkpoint therapy and therapeutic vaccines for PC. Moreover, immunotherapy has already been used in clinical trials for other malignant neoplasms, and positive clinical outcomes were observed in colon, renal, and lung cancer and in metastatic melanoma [6]. A key to the successful management of metastatic castrate-resistant prostate cancer (CRPC) is to understand the complexity of tumor cells and their interactions with the surrounding microenvironment, in particular with infiltrating macrophages and lymphocytes [7]. During the course of the disease, neoplastic cells develop a mechanism of an immune escape and develop resistance to proapoptotic signals, for example, by blocking immune checkpoints in the PD-1, PD-L1, PD-L2, and CTLA-4 axes [8].

## 2. The Evolving Role of Immunotherapy in Prostate Cancer

### 2.1. Prostate Cancer Vaccines

In contrast to other solid tumors, cancer of the prostate was seen as an inflammatory disease for a long time. Recent studies on murine models revealed that chronic inflammation is preceded by endothelial changes that allow immune cell extravasation. Many studies have evaluated the relationship between specific immune cells and prostate cancer. In prostate cancer, we have learned to use significant amounts of vaccines, but they are still behind the results observed in other solid tumors such as melanoma, bladder and kidney cancer, and non-small-cell lung cancer. Currently, several vaccines for prostate cancer are available; however, most of them fail to meet expectations.

PC cells usually proliferate slowly, providing the time needed to elicit an immune response, even in patients with advanced disease. Hence, the PC represents an ideal target for cancer vaccines [9] (Table 2). Sipuleucel-T is an autologous vaccine in which the patient's peripheral blood mononuclear cells are retrieved via leukapheresis [3]. Vaccine's target is prostatic acid phosphatase (PAP), a glycoprotein enzyme synthesized in the prostate epithelium that significantly increases as cancer progresses. PAP is elevated in patients with bone metastasis and correlates with poor prognosis [10]. According to the phase III clinical trial known as Immunotherapy for Prostate Adenocarcinoma Treatment (IMPACT), treatment with sipuleucel-T resulted in a 4.1-month overall survival (OS) benefit and a 22% relative risk reduction of mortality in patients with metastatic CRPC [11]. Data from IMPACT also revealed that the greatest benefit occurs in patients with a lower disease burden [9, 12] indicating the importance of early screening and diagnosis of PCa. Sipuleucel-T is approved by the Food and Drug Administration (FDA); however, the treatment is currently cost-prohibitive [13]. Despite the survival benefits, only minimal antineoplastic responses were observed. It turned out that PC compared to melanoma responds to checkpoint inhibitors in a totally different manner. Vaccines seem to have a subtle impact on immunological microenvironment. There is evidence that antigen-specific B and antigen-specific T cell responses may be generated early, for example, after the first infusion, and can be restimulated in vitro. Many cytokines are involved in T cell activation including IL-2, IL-4, IL-5, IL-6, IL-10, IL-13, IL-17, interferon gamma (IFN-*γ*), and tumor necrosis factor-alpha. All of them can be detected in cell culture fluids after the second and third signal [14, 15].

Prostvac-VF is a viral-based vaccine based on a combination of two viral vectors, vaccinia that is a potent immunologic priming agent and fowlpox that is used as a boosting agent [9]. Each vector encodes for PSA and three immune costimulatory molecules including intracellular adhesion molecule 1, costimulatory molecule for T cells (B7–1), and lymphocyte function-associated antigen 3 [16]. The virus infects antigen-presenting cells (APCs), promoting cell surface protein expression and interaction with T cells, in consequence facilitating targeted immune response and cell-mediated tumor cell destruction [17]. In the phase II clinical trial, Prostvac-VF was well tolerated and improved OS compared with control vectors (25.1 months versus 16.6 months) in patients with minimally symptomatic CRPC [17]. Patients with aggressive or end-stage disease exhibited lower benefits [17, 18]. In contrast to preparation of sipuleucel-T, this construct is based on the inherent immunogenicity of the vaccinia virus. Induced T cell response directed against PSA can have other antigens that can activate other T cells. Results of phase I were encouraging for a phase II study, suggesting that the benefits of survival are comparable with the values of sipuleucel-T. However, the results of phase III trials are eagerly awaited [17]. GVAX is an allogeneic whole cell-based prostate cancer vaccine. In this approach, autologous or allogeneic tumor cells are genetically modified to bear GM-CSF [3]. GM-CSF induces the recruitment of APCs invoking a cascade of immune responses [9]. The whole tumor cell is used as an antigen that consequently facilitates both humoral and cellular immune responses. Although phase I and II studies confirmed clinical activity and safety, phase III was stopped due to increased mortality and futility analysis [3, 9].

DCVAC/PCa is an autologous dendritic cell-based vaccine composed of poly I:C-activated DCs pulsed with killed LNCaP prostate cancer cell line. Phase I and II trials showed that chemoimmunotherapy combined with DCVAC and docetaxel resulted in a 7.2-month OS benefit with no significant complications [19]. Currently, a phase III clinical trial (NCT02111577) is being conducted to evaluate the efficacy and safety of DCVAC/PCa versus placebo in men with metastatic CRPC eligible for first-line chemotherapy.

DNA-based vaccines consist of genetically engineered DNA containing the coding sequence of a targeted antigen. This sequence can be taken up by cells which subsequently express the genes that induce an immune response [20]. Phase I trials have been done targeting various tumor-associated antigens, including PSA, PSMA, PAP, and the cancer-testis antigen NY-ESO-1. Little clinical efficacy has been demonstrated to date; however, most trials have demonstrated immunologic activity [21].

## 3. Novel Therapies in Castration-Resistant Prostate Cancer: The Blockade of Immune Checkpoints

### 3.1. CTLA-4-Based Immunotherapy

The first monoclonal antibody (ipilimumab, Yervoy™) directed against the control molecule, CTLA-4 [22], was approved for melanoma to improve survival and increase antitumor efficacy. CTLA-4 is a protein receptor that belongs to the immune checkpoints. It downregulates immune responses. CTLA-4 appears on the surface of T lymphocytes activated by contact with the antigen and acts to inhibit further lymphocyte response. T cells require two signals to become fully activated. CD28 and CTLA-4 are T cell receptors that play a decisive role in initial activation and subsequent control of cellular immunity. CD28 transmits a stimulatory signal to T cells. CTLA-4 is homologous to CD28. Both molecules bind to B7 on APCs. CTLA-4 binds to B7 with a greater affinity and avidity than CD28 thus enabling it to outcompete CD28 for its ligands. Ipilimumab is a fully human monoclonal antibody that decreases the binding of CTLA-4 to B7, which results in enhanced antitumor immunity [23]. A phase III trial in which men with CRPC that had progressed after docetaxel chemotherapy were treated with radiation therapy to a bone metastasis followed by either ipilimumab or placebo indicated that ipilimumab can prolong median OS in a select subset of patients lacking visceral disease and with favorable laboratory values [24]. However, patients who did not receive docetaxel did not achieve overall survival benefit, but it was suggested that patients with visceral metastases had poorer prognosis [22]. Combining ipilimumab with prostate cancer vaccines appears even more beneficial for the patients [24, 25].

### 3.2. Roles of the PD-1, PD-L1, and PD-L2 Pathway in Healthy Hosts

PD-1 (programmed cell death receptor-1) (also known as CD279) belongs to the CD28 (or B7) family and is coded by the PDCD-1 gene (programmed cell death genes) which is localized on chromosome 2 (2q37). PD-1 is a type I transmembrane glycoprotein composed of 288 amino acids [26]. PD-1 is expressed on the cellular surface of activated T cells (cytotoxic T lymphocytes (CTLs)) and B cells and on the activated monocytes, dendritic cells and natural killer (NK) cells, NKT cells, dendritic cells (DCs), and macrophages [27]. PD-1 is responsible for regulating immune responses and programmed cell death. PD-1 participates in induction and supports peripheral T cell immunity [28]. The crucial role of this immune checkpoint receptor in the inflammatory process is to reduce T cell activity in peripheral tissue, preventing autoimmunity [29].

PD-1 can bind to one of its two ligands, programmed cell death protein ligand 1 (PD-L1) or programmed cell death protein ligand 2 (PD-L2). Both of them are expressed on the surface of tumor cells and correlate with patient prognosis [30]. PD-1 signaling inhibits allogeneic activation of T cells and may promote inducible regulation of T cell development. Furthermore, it influences several control points of the cell cycle [31–33]. Ligands for the PD-1 receptor are located on the surface of APCs and target cells (tumor cells). Interaction of PD-L1 or PD-L2 with the PD-1 antigen located on the surface of lymphocyte causes inhibition of their activity and leads to the blockade of immune response [34].

Recent studies reported successful use of an anti-PD-1 antibody in the treatment of advanced melanoma (FDA-approved pembrolizumab in September 2014) and metastatic melanoma (FDA-approved nivolumab in December 2014) [35, 36]. Moreover, nivolumab was approved for metastatic or advanced non-small-cell lung cancer in March 2015 [37]. There are only two clinical trials with anti-PD-L1 and anti-PD-L2 for prostate cancer. Pembrolizumab is being investigated in a phase II study in metastatic CRPC after androgen deprivation therapy (NCT02312557) [38]. Pidilizumab in combination with sipuleucel-T and cyclophosphamide is being studied in metastatic CRPC (NCT01420965) [39] (Table 3).

### 3.3. Cytokines in Prostate Cancer Immunotherapy

Stimulation of the immune system by affecting the cytokines may result in a strong antitumor immunity [40]. It is associated with the activity of innate and adaptive immune system [41]. Studies on PC demonstrated elevated levels of numerous interleukins such as IL-1*α*, IL-2, IL-4, IL-6, IL-7, IL-11, IL-12, IL-15, IL-17, IL-18, IL-27, and IL-35 [42–51]. The levels of interleukins usually correlate with the progression of PC including metastasis [42–51]. Recent studies focus on the possibility of blocking interleukins or their receptors. Cheng et al. showed that mesenchymal stem cells pretreated with IL-1*α* promoted the growth of prostate RM-1 mouse cancer cell line [42]. Dieli et al. in a phase I clinical trial investigated implications of the *γδ* T cell agonist zoledronate with or without IL-2 for metastatic hormone-resistant prostate cancer. Most patients who received only zoledronate had progressive clinical deterioration, while a combination with IL-2 induced better clinical response [44]. Mackiewicz et al. demonstrated that vaccination with TRAMP-H6 (vaccines modified with hyper-IL-6) and TRAMP-H11 (vaccines modified with hyper-IL-11) extends OS of mice with PC [45]. Recent studies indicate that the inflammatory process initiated by IL-17 may induce the progression of PC [52–54]. Yang et al. showed that expression of PD-1 and its ligands was higher in IL-17rc wild-type mouse PCs than IL-17rc-knockout mouse PCs. Furthermore, PD-1 expression was found primarily in the infiltrating inflammatory cells, while that of PD-L1 and PD-L2 was found in the neoplastic epithelial cells. It is suggested that elevated expression of PD-1 and its ligands promotes murine PC progression [7].

### 3.4. The Multitude of Different Potential Treatment Combinations for Prostate Cancer

The heterogeneity of prostate cancer, treatment resistance, and the growing need for individual therapy guide the latest research into combining different approaches. Although various drugs are available now in clinical practice, the potential toxicity resulting from their interactions has to be assessed [55, 56].

There are ongoing studies on combination vaccines with androgen deprivation therapy (ADT) and radiotherapy (RT). RT induces vaccination on T cells by releasing tumor antigens and soluble proinflammatory mediators. ADT, on the other hand, promotes lymphopoiesis, immune cell trafficking, and tumor penetration. Both strategies may be used in conjunction with immunotherapy. Clearly, maximum synergy can be achieved by thoroughly investigating each intervention at the exact phase of the immune response induced by therapy. The immune modulation is rather difficult and depends on many factors such as ADT type, RT strategy (type, dose, and duration), and administered immunotherapeutic agent. In a pilot study of intratumoral DC administration, patients remaining in the therapy of androgen suppression (GnRH agonist and bicalutamide) underwent external beam radiotherapy (EBRT) [55]. One patient had transient preexisting T cell responses to PSA, PSMA, and Her2/neu. Larger studies are needed to determine the optimal use of DC-based immunotherapy with RT-induced apoptosis and inflammatory responses [57].

A multicenter randomized phase II study tests active DCVAC/PCa cell immunotherapy in patients with localized high-risk prostate cancer after primary RT. The purpose of this study is to determine whether DCVAC/PCa can improve PSA progression times. This study is ongoing. The estimated completion date for the study is scheduled for September 2018 [58].

Another randomized phase II clinical trial investigates the role of ^153^Sm-EDTMP (Quadramet) with or without a PSA/TRICOM vaccine in men with androgen-insensitive metastatic prostate cancer. Patients treated with both PSA-TRICOM and ^153^Sm-EDTMP were found to have an increase in PSA-specific T cell lymphocytes and lower levels of circulating myeloid-derived suppressor cell (MDSC) subgroups compared to patients in the ^153^Sm-EDTMP alone after 60 days of therapy. Although a statistically significant difference in overall survival was observed, patients receiving ^153^Sm-EDTMP and PSA-TRICOM experienced more than twice the disease progression compared to those receiving ^153^Sm-EDTMP alone (3.7 months versus 1.7 months, resp.). This vaccination strategy resulted in a strong immunological response in tumor biopsy, with a marked prolongation of PSA doubling time [59].

The alternative approach addresses CTLA-4, PD-1, PD-L1, and PD-L2. McNeel et al.6 tested tremelimumab in combination with bicalutamide in biochemically recurrent PC after local therapy. Eleven patients were enrolled and completed at least 1 year of follow-up. Monotherapy of ipilimumab resulted in decreased PSA level; however, the primary endpoint was not reached [60]. The early results of phase II studies on pembrolizumab in combination with enzalutamide showed a complete PSA response in 3/10 patients. Tumor regression indicates a great potential for a combination of immune checkpoint blockades for PC [61].

## 4. Conclusions

Science's editors have chosen cancer immunotherapy as the breakthrough of the year for 2013 [62]. Neoplastic cells naturally escape from the control of the immune system, and the main goal of immune therapy is to bring the control back.

Satisfying outcomes after treatment of advanced melanoma and lung cancer suggest a great potential of immunotherapy as an approach for other tumors' treatment, especially in patients primarily introduced to palliative care.

After initial clinical trials, immunotherapy seems to have different side effects than chemotherapy [63, 64]. Prostate cancer was the first neoplasm in which a specific vaccine significantly improved survival. Preliminary studies on new drugs blocking the immune checkpoints in CRPC indicate that it might be a solution for these patients. There is a tremendous potential for synergistic combinations of immunotherapy with conventional cancer treatments [65]. A combination of several drugs or methods can be a key in radical treatment of metastatic prostate cancer as demonstrated by preliminary studies. We definitely need more studies to clearly define the role of immunotherapy in the treatment of advanced prostate cancer.

## Figures and Tables

**Table 1:** Types of cancer immunotherapy.

Blockade of immune checkpoint	Cytokines	Therapeutic vaccines
Disrupts signals that allow cancer immune evasion	Direct the immune system against cancer cells	Enhance host's natural immune response against cancer

**Table 2:** Prostate cancer vaccines.

Drug	Agent description-based vaccine
Sipuleucel-T	Targets prostatic acid phosphatase (PAP)
Prostvac-VF	Fowlpox virus
GVAX	Expressing GM-CSF
DCVAC/PCa	Poly I:C

**Table 3:** The blockade of immune checkpoints in prostate cancer.

Drug	Agent description
Ipilimumab	The inhibition of CTLA-4
Pembrolizumab (previously known as MK-3475 and lambrolizumab)	The inhibition of PD-1
Pidilizumab	The inhibition of PD-1
